# Supplementary material for: Test performance of lateral flow rapid antigen tests for COVID-19 in Welsh adult care home staff using routine surveillance data
Source: PLoS One. 2023 Aug 23;18(8):e0290406. doi: 10.1371/journal.pone.0290406 (PMC10446167; doi:10.1371/journal.pone.0290406)
Supplement: Attachment — Submitted filename: PONE-D-23-02428.pdf [file pone.0290406.s002.pdf]

PLOS ONE

Test performance of lateral flow rapid antigen tests for COVID-19 in Welsh adult care home staff using routine surveillance data

--Manuscript Draft--

**Manuscript Number:**	PONE-D-23-02428
**Article Type:**	Research Article
**Full Title:**	Test performance of lateral flow rapid antigen tests for COVID-19 in Welsh adult care home staff using routine surveillance data
**Short Title:**	Test performance of lateral flow rapid antigen tests for COVID-19 in Welsh adult care home staff
**Corresponding Author:**	Craig John Hogg<br>Public Health Wales<br>Cardiff, Wales UNITED KINGDOM
**Keywords:**	COVID-19; test performance; rapid antigen testing; sensitivity and specificity; Coronavirus; SARS-CoV-2; Surveillance
**Abstract:**	Background: Lateral flow tests (LFTs) have been used to screen for SARS-CoV2 in Wales since January 2021. Between May and August 2021, adult care home staff policy was for biweekly Innova LFT and weekly Polymerase Chain Reaction (PCR) testing while asymptomatic. We estimated test performance of LFTs conducted in adult care home staff using PCR tests as a gold standard.<br>Methods: Test results from surveillance data were matched by individual where both LFT and PCR were taken on the same day. We calculated sensitivity, specificity, positive and negative predictive values, and agreement using Matthew's correlation coefficient. Ct values of positive PCR results were compared by matched LFT result. Analysis was conducted using R v4.1.3.<br>Results: We analysed 115,593 test pairs, 499 (0.43%) of which were PCR positive. Median age was 48 (IQR: 22) and 85.00% of the study population were female. Test result agreement was 99.59% (95%CI 99.55-99.63; MCC: 0.38, p<0.001). Sensitivity and specificity were 25.65% (95%CI 22.02-29.67) and 99.91% (95%CI 99.89-99.93), respectively. PPV was 55.90% (95%CI 49.42-62.17) and NPV was 99.68% (95%CI 99.64-99.71). Crude Ct values were significantly lower in positive PCR tests matched to a positive LFT compared to a negative LFT.<br>Conclusions: Specificity and negative predictive value were high in an asymptomatic population of care home staff indicating this test is an effective tool to rule in SARS-CoV-2 infection. Positive predictive value results are lower than existing literature yet should be considered in light of the asymptomatic study population and low prevalence (under 1%) at the time most of these tests were conducted. These results suggest that lateral flow tests should not be used to rule out infection. LFTs were also unsatisfactory as a tool to rule in infection due to low positive predictive value in this sample, but performance improved at times of higher prevalence during the study.
**Order of Authors:**	Craig Hogg
	Sian Boots
	Daniel Howorth
	Christopher Williams
	Margaret Heginbothom
	Jane Salmon
	Robin Howe
**Additional Information:**	
**Question**	**Response**
**Financial Disclosure**	The author(s) received no specific funding for this work.

Powered by Editorial Manager® and ProduXion Manager® from Aries Systems Corporation

Enter a financial disclosure statement that describes the sources of funding for the work included in this submission. Review the submission guidelines for detailed requirements. View published research articles from *PLOS ONE* for specific examples.<br><br>This statement is required for submission and **will appear in the published article** if the submission is accepted. Please make sure it is accurate.<br><br>**Unfunded studies**<br>Enter: *The author(s) received no specific funding for this work.*<br><br>**Funded studies**<br>Enter a statement with the following details:<br>• Initials of the authors who received each award<br>• Grant numbers awarded to each author<br>• The full name of each funder<br>• URL of each funder website<br>• Did the sponsors or funders play any role in the study design, data collection and analysis, decision to publish, or preparation of the manuscript?<br>• **NO** - Include this sentence at the end of your statement: *The funders had no role in study design, data collection and analysis, decision to publish, or preparation of the manuscript.*<br>• **YES** - Specify the role(s) played.<br><br>* typeset	
**Competing Interests**<br><br>Use the instructions below to enter a competing interest statement for this submission. On behalf of all authors, disclose any competing interests that could be perceived to bias this work—acknowledging all financial support and any other relevant financial or non-financial competing interests.<br><br>This statement is required for submission and **will appear in the published article** if the submission is accepted. Please make	The authors have declared that no competing interests exist.

Powered by Editorial Manager® and ProduXion Manager® from Aries Systems Corporation

sure it is accurate and that any funding sources listed in your Funding Information later in the submission form are also declared in your Financial Disclosure statement.<br><br>View published research articles from *PLOS ONE* for specific examples.<br><br>**NO authors have competing interests**<br><br>Enter: *The authors have declared that no competing interests exist.*<br><br>**Authors with competing interests**<br><br>Enter competing interest details beginning with this statement:<br><br>*I have read the journal's policy and the authors of this manuscript have the following competing interests: [insert competing interests here]*<br><br>* typeset	
**Ethics Statement**<br><br>Enter an ethics statement for this submission. This statement is required if the study involved:<br><br>• Human participants<br>• Human specimens or tissue<br>• Vertebrate animals or cephalopods<br>• Vertebrate embryos or tissues<br>• Field research<br><br>Write "N/A" if the submission does not require an ethics statement.<br><br>General guidance is provided below. Consult the submission guidelines for detailed instructions. **Make sure that all information entered here is included in the Methods section of the manuscript.**	Public Health Wales was established under The Public Health Wales National Health Service Trust (Establishment) Order 2009. Its functions include the provision and management of a range of public health, health protection, healthcare improvement, health advisory, child protection and microbiological laboratory services and services relating to the surveillance, prevention and control of communicable diseases. This work falls under the establishment order and therefore no further NHS research permissions were required. Data were held and processed under Public Health Wales information governance arrangements in compliance with the Data Protection Act, Caldicott Principles and Public Health Wales guidance on the release of small numbers. All methods were performed in accordance with the relevant guidelines and regulations. No data identifying protected characteristics of an individual were released outside Public Health Wales. Data used in the study was anonymised prior to analysis.

**Format for specific study types**<br><br>**Human Subject Research (involving human participants and/or tissue)**<br>• Give the name of the institutional review board or ethics committee that approved the study<br>• Include the approval number and/or a statement indicating approval of this research<br>• Indicate the form of consent obtained (written/oral) or the reason that consent was not obtained (e.g. the data were analyzed anonymously)<br><br>**Animal Research (involving vertebrate animals, embryos or tissues)**<br>• Provide the name of the Institutional Animal Care and Use Committee (IACUC) or other relevant ethics board that reviewed the study protocol, and indicate whether they approved this research or granted a formal waiver of ethical approval<br>• Include an approval number if one was obtained<br>• If the study involved *non-human primates*, add *additional details* about animal welfare and steps taken to ameliorate suffering<br>• If anesthesia, euthanasia, or any kind of animal sacrifice is part of the study, include briefly which substances and/or methods were applied<br><br>**Field Research**<br><br>Include the following details if this study involves the collection of plant, animal, or other materials from a natural setting:<br>• Field permit number<br>• Name of the institution or relevant body that granted permission	
**Data Availability**<br><br>Authors are required to make all data underlying the findings described fully available, without restriction, and from the time of publication. PLOS allows rare exceptions to address legal and ethical concerns. See the PLOS Data Policy and FAQ for detailed information.	No - some restrictions will apply

Powered by Editorial Manager® and ProduXion Manager® from Aries Systems Corporation

A Data Availability Statement describing where the data can be found is required at submission. Your answers to this question constitute the Data Availability Statement and **will be published in the article**, if accepted.<br><br>**Important:** Stating 'data available on request from the author' is not sufficient. If your data are only available upon request, select 'No' for the first question and explain your exceptional situation in the text box.<br><br>Do the authors confirm that all data underlying the findings described in their manuscript are fully available without restriction?	
**Describe where the data may be found in full sentences. If you are copying our sample text, replace any instances of XXX with the appropriate details.**<br><br>• If the data are **held or will be held in a public repository**, include URLs, accession numbers or DOIs. If this information will only be available after acceptance, indicate this by ticking the box below. For example: *All XXX files are available from the XXX database (accession number(s) XXX, XXX.).*<br>• If the data are all contained **within the manuscript and/or Supporting Information files**, enter the following: *All relevant data are within the manuscript and its Supporting Information files.*<br>• If neither of these applies but you are able to provide **details of access elsewhere**, with or without limitations, please do so. For example:<br><br>*Data cannot be shared publicly because of [XXX]. Data are available from the XXX Institutional Data Access / Ethics Committee (contact via XXX) for researchers who meet the criteria for access to confidential data.*<br><br>*The data underlying the results presented in the study are available from (include the name of the third party*	Full data cannot be shared publicly as it concerns individual human participant data prior to test pair creation.<br>The anonymised data underlying the results presented in the study are within the manuscript and its Supporting Information files.

and contact information or URL).<br>• This text is appropriate if the data are owned by a third party and authors do not have permission to share the data.<br><br>* typeset	
Additional data availability information:	

Powered by Editorial Manager® and ProduXion Manager® from Aries Systems Corporation

Manuscript

Click here to access/download;Manuscript;LFT Test Performance Care Home Staff Final Draft.docx

Test performance of lateral flow rapid antigen tests for COVID-19 in Welsh adult care home staff using routine surveillance data

Craig Hogg[1 ¶ *], Sian Boots[1¶], Daniel Howorth[1], Christopher Williams[1], Margaret Heginbothom[1], Jane Salmon[1], Robin Howe[2]

[1]Communicable Disease Surveillance Centre, Public Health Wales, Cardiff, Wales, UK

[1]Public Health Wales Microbiology, Cardiff, Wales, UK

[*] Corresponding Author

E-mail: Craig.Hogg@wales.nhs.uk

[¶] CH and SB are Joint Senior Authors

Author Contributions

CH contributed to the conception and implementation of the main data processing and analysis and writing the manuscript. SB contributed to the conception and implementation of the main data processing and analysis and writing the manuscript. DH contributed to data processing and writing the manuscript. CW contributed to formal analysis and review of the manuscript. MH contributed to conception of the overall study, project supervision and review of the manuscript. JS contributed to conception of the overall study, project supervision and review of the manuscript. RH contributed to conception of the overall study and review of the manuscript. The author(s) read and approved the final manuscript.

Abstract

Background

Lateral flow tests (LFTs) have been used to screen for SARS-CoV2 in Wales since January 2021. Between May and August 2021, adult care home staff policy was for biweekly Innova LFT and weekly Polymerase Chain Reaction (PCR) testing while asymptomatic. We estimated test performance of LFTs conducted in adult care home staff using PCR tests as a gold standard.

Methods

Test results from surveillance data were matched by individual where both LFT and PCR were taken on the same day. We calculated sensitivity, specificity, positive and negative predictive values, and agreement using Matthew’s correlation coefficient. Ct values of positive PCR results were compared by matched LFT result. Analysis was conducted using R v4.1.3.

Results

We analysed 115,593 test pairs, 499 (0.43%) of which were PCR positive. Median age was 48 (IQR: 22) and 85.00% of the study population were female. Test result agreement was 99.59% (95%CI 99.55-99.63; MCC: 0.38, $p<0.001$). Sensitivity and specificity were 25.65% (95%CI 22.02-29.67) and 99.91% (95%CI 99.89-99.93), respectively. PPV was 55.90% (95%CI 49.42-62.17) and NPV was 99.68% (95%CI 99.64-99.71). Crude Ct values were significantly lower in positive PCR tests matched to a positive LFT compared to a negative LFT.

Conclusions

Specificity and negative predictive value were high in an asymptomatic population of care home staff indicating this test is an effective tool to rule in SARS-CoV-2 infection. Positive predictive value results are lower than existing literature yet should be considered in light of the asymptomatic study population and low prevalence (under 1%) at the time most of these tests were conducted. These results suggest that lateral flow tests should not be used to rule out infection. LFTs were also unsatisfactory as a tool to rule in infection due to low positive predictive value in this sample, but performance improved at times of higher prevalence during the study.

Background

On the 11th March 2020 the World Health Organisation declared a coronavirus disease-19 (COVID-19) pandemic as a result of a newly identified Severe Acute Respiratory Syndrome-related coronavirus (SARS-CoV-2) [1,2]. Worldwide epidemiological surveillance systems to monitor COVID-19 ~~have~~ developed rapidly as has the development of rapid non-invasive antigen lateral flow tests (LFTs) designed to detect SARS-COV-2. Whilst polymerase chain reaction tests (PCRs) are able to pick up lower levels of SARS-COV-2 than LFTs, rapid antigen testing can provide results quickly and cost effectively [3.4]. They can therefore be a useful early detection tool for COVID-19 case clusters in asymptomatic individuals [5]. However, there has been much debate as to whether LFTs exhibit adequate performance to meet the needs of a mass-surveillance and screening system [6].

In January 2021, the Welsh Government launched a COVID-19 testing strategy that included the use of LFTs to aid in preventing the transmission of COVID-19 across Wales [7]. A part of this strategy involved the use of LFTs in closed settings to reduce the risk of transmission from staff to care home residents. In these closed settings, asymptomatic staff members were advised to undertake LFTs twice or more per week [8].

In January 2022, policy change removed the requirement to confirm a positive LFD results with a PCR test [9]. In April 2022, policy identified LFTs as the primary testing route for the symptomatic diagnosis of COVID-19. These rapid changes in policy increased the need to evaluate this test. Interpretation of this evaluation will be affected by restrictions and management of cases deemed to be 'false positive' [10], and the use of LFTs as a routine screening tool [11].

With the data generated as a result of the implementation of the Testing Strategy for Wales, we aim in this paper to measure the sensitivity and specificity of LFTs against PCRs for those

tests taken in Adult Care Home settings in Wales during the period 1st May 2021 – 31st August 2021.

Methods

Study Population

Individuals were included in the study population where their postcode of residence was in Wales, and they were of working age (between 18 and 67 years old at time of first test within the timeframe). Both the LFT result and PCR result must be positive or negative (not void), and conducted between 1st May 2021 and 31st August 2021. From this study population, we then only included individuals whose LFT record was associated with an adult care home, and who identified as a member of staff in the care home. Following guidance, symptomatic individuals should book a PCR test in the first instance, therefore this study population is presumed to be primarily asymptomatic.

Data Sources

Lateral flow testing activity data for Wales was sourced from the UK Government Lateral Flow Testing Portal, collated by NHS Digital, and supplied to Public Health Wales (PHW) via a data feed and a set of SQL views from Digital Health and Care Wales (DHCW).

PCR testing data was sourced from NHS and lighthouse laboratories across Wales and supplied to PHW through a data feed from DHCW.

A list of adult care home unique organisation numbers (UONs) and UON groups in line with the Testing Strategy for Wales was provided to PHW and DHCW by the Welsh Government.

All data was collected as part of routine surveillance and management of COVID-19. Patient identifiable information was anonymised when necessary and held securely on the NHS Wales Network.

Test Matching

Lateral flow test records and PCR test records were matched on a 1:1 basis a unique reference code where both LFT and PCR were taken on the same day. Where an individual conducted multiple tests of the same type on the same day, the earliest lateral flow with a valid result was matched with the earliest PCR with a valid result conducted on that day. Subsequent LFTs were matched with subsequent PCR tests with a preference to the next earliest test.

Lateral Flow Assay

Lateral flow assay tests were taken by the individual using Innova test kits. Presuming guidance was followed correctly, self-administered combined nose and throat swabs were sampled and interpreted at 30 minutes. Results were then self-reported in conjunction with contact and demographic information to an online portal hosted by the UK Government.

PCR Assay

RT-PCR was used as the ‘gold standard’ in this analysis. No classification was recorded regarding whether the swab was conducted by the individual or a trained professional. Combined nose and throat swabs or dry throat swabs were sampled and RT-PCR was then conducted. Results were graded and authorised by trained laboratory staff according to manufacturer definitions and standard operating procedures (Welsh Government TAC, 2020). Sample processing equipment specification was likely to vary by time and test processing location and therefore was not identical in every test. Ct values for positive PCR results were not available for stratified analysis.

Statistical Analysis

Specificity, sensitivity, and predictive values were calculated using the RT-PCR results as gold standard. Where applicable, 95% Confidence Intervals for proportions were calculated

using the Wilson method. Matthew's correlation coefficient (MCC) was used to evaluate the level of agreement between tests. Ct values were extracted where available for PCR results and were compared between LFT positive and LFT negative groups, using Mann-Whitney to test for significance. Statistical analyses were performed using R v4.1.0.

Ethics

Public Health Wales was established under The Public Health Wales National Health Service Trust (Establishment) Order 2009. Its functions include the provision and management of a range of public health, health protection, healthcare improvement, health advisory, child protection and microbiological laboratory services and services relating to the surveillance, prevention and control of communicable diseases. This work falls under the establishment order and therefore no further NHS research permissions were required. Data were held and processed under Public Health Wales information governance arrangements in compliance with the Data Protection Act, Caldicott Principles and Public Health Wales guidance on the release of small numbers. All methods were performed in accordance with the relevant guidelines and regulations. No data identifying protected characteristics of an individual were released outside Public Health Wales. Data used in the study was anonymised prior to analysis.

Results

Characteristics of the study population

Characteristics of the selected study population (n=115,593) are shown in Table 1. These demographics are shown at test level and include repeated testing by individuals.

Table 1. Summary of characteristics of test records which contributed toward the study population.

Total *N* (Valid LFTs with a linked valid PCR)	115,593
Positive PCR (% (*n*))	0.43% (499)
Positive LFT (% (*n*))	0.20% (229)
Sex (%F (*n*))[a]	85.00% (98,741)
Age (Median (IQR))	48 (22)

[a] Sex was unknown or unspecified in 116 records

Test Results

In these 115,593 linked test records, 0.43% of PCR results were positive (n=499) and 99.57% were negative (n=115,094), whilst 0.20% of LFT results were Positive (n=229) and 99.80% were negative (n=115,364) (Table 2).

Table 2. Summary of Innova LFT results compared to PCR.

	**Innova Lateral Flow**		
**RT-PCR**	Positive	Negative	TOTAL
Positive	128	371	499
Negative	101	114,993	115,094
TOTAL	229	115,364	115,593

Agreement between groups was calculated to be 99.59% (MCC: 0.38; 95%CI 99.55, 99.63). Of the 115,593 tests in the study population, 472 matched tests did not agree. A total of 78.60% (n=371) of these cases had a negative LFT followed by a Positive PCR ("False Negatives") whilst 21.40% (n=101) had a positive LFT followed by a negative PCR ("False Positives").

Test Performance

Using this data, overall sensitivity of the lateral flow devices was 25.65% (95%CI 22.02-29.67) and specificity was 99.91% (95%CI 99.89, 99.93). Across the entire study period,

Negative Predictive Value (NPV) was estimated to be 99.68% (95%CI 99.64, 99.71), whilst Positive Predictive Value (PPV) was estimated to be 55.90% (95%CI 49.42, 62.17) (Table 3). PPV however varied over time due to changing prevalence during the study period, ranging from a minimum (non-zero) estimate of 22.22% to a maximum of 85.19% (Fig 1). [13]

Table 3. Test performance measures calculated for Innova lateral flow device in asymptomatic care home staff.

Measure	Estimate (95% CI)
Sensitivity (95%CI)	25.65% (22.02, 29.67)
Specificity (95%CI)	99.91% (99.89, 99.93)
Positive Predictive Value (95%CI)	55.90% (49.42, 62.17)
Negative Predictive Value (95%CI)	99.68% (99.64, 99.71)
Agreement (95%CI; MCC)	99.59% (99.55, 99.63; 0.38)
Agreement *p* value[a]	<0.001***

[a] Fisher's exact test

Fig 1. Prevalence Estimate comparison (left axis) and Positive Predictive Value (right axis) by week. Data excluded for weeks 17 and 35 due to incomplete weekly data.

PCR Ct Values

Ct values were available for 51 (10.2%) of positive PCR results included in the study. We tested the distribution of Ct results and found them not to be normally distributed (Shapiro-Wilk: W=0.904, p < 0.001***). Control target values were available for less than half of these results (45.1%) so we did not proceed to normalisation of minimum Ct values.

Wilcoxon rank sum test yielded a p value < 0.001, suggesting a significant difference in crude minimum Ct values between matched LFT result groups (Fig 2).

Fig 2. Minimum target Ct value, by matched LFT result. Labels show median of each group and statistical difference between groups.

Discussion

We believe this at time of publication to be the first study of lateral flow test performance using self-reported results from the UK Government online portal demonstrating real-world test performance estimates. This study has identified that Innova lateral flow testing kits supplied as part of the Testing Strategy for Wales yielded a high specificity reaching above 99%, consistent with previously published literature and indicating this test could be an effective tool to rule in SARS-CoV-2 infection where the test result is positive due to the very low false positive rate. In addition, PPV results are much lower than existing literature, yet should be considered in light of the asymptomatic study population and low prevalence (under 1%) during this study. This value increases notably with increased prevalence, further supporting the use of this test to rule in infection. Sensitivity analysis however yielded a lower sensitivity than expected, with an overall estimate of 25.65%. Due to this, this analysis does not support use of lateral flow tests as a reliable diagnosis platform as the test cannot reliably rule out infection, particular at times of low prevalence where NPV will be reduced. This contrasts with the results of a systematic review by Mistry *et al* [14], which calculated a pooled sensitivity estimate of 78.68% in Innova test kits.

Previous studies have demonstrated lower sensitivity estimates in self-swabbing samples compared to those in studies where the test was undertaken by a trained professional [14]. However the difference shown by Mistry et al was not significant and their estimates were not as low as the results found in this analysis. A likely major factor contributing to this is that the testing programme is targeted towards asymptomatic individuals, which has been shown to yield lower sensitivity estimates than a symptomatic population [15].

Viral load is also a possible contributing factor to the likelihood of detection in lateral flow devices [16]. However, there is a lack of evidence supporting a difference in viral load between symptomatic and asymptomatic cases [17,18], therefore further research in this area would be of benefit to understand the influence this may have on test performance. Differences in Ct values between matched LFT results in these analyses suggest a statistically significant relationship between test agreement and Ct value as a proxy for viral load. However, limited numbers of available Ct values in this case mean further work is required to understand this relationship. A further limitation is that less than half of available Ct values had control values which were able to be used to analyse normalised Ct results. Whilst preliminary analysis of the available normalised values showed similar trends, a more robust approach would be required to draw any meaningful conclusion from such analysis.

Research has shown a person may shed SARS-COV-2 for up 17 days, however live virus was only found within 9 days [19,20]. Therefore, as a result of the high sensitivity of PCRs, they could yield a positive result for a longer period of time compared to a LFT which only detect higher viral loads, creating a narrow time period where both results would give a positive result [20]. There is also variability in sensitivity and specificity of PCR tests which may differ depending on the manufacturer of the assay or machine used. This may influence these test performance characteristics due to this variance in the PCR as ‘gold standard’ reference test.

Due to the repeated testing in our study population, it is possible that previously PCR positive individuals may continue to test positive by PCR where public testing guidance would advise against repeated testing in this post-infectious period [21,22]. As Lateral Flow Tests would be unlikely to identify infection in this period [20], this aspect of the study population's testing regime may negatively impact the sensitivity estimates in this analysis. There may also be implications to the calculation of prevalence in this scenario, and therefore may partially account for the Positive Predictive Value of 55.90%. However, comparison with ONS Coronavirus Infection Survey [13,23] showed this data displayed similar prevalence to the overall Welsh population at the time these tests were conducted (Fig 1), further supporting the reliability of the PPV estimate given in this paper.

Poor sampling technique in a self-testing regime could be a contributing factor to the low sensitivity results produced in this analysis. However, a study by Amoo et al. [24] found that self-sampling for qPCR analysis was reliable and yielded a high agreement percentage compared with samples collected by healthcare workers. Agreement in this study population was 99.59%, however this is difficult to interpret in light of the data skew towards negative-negative matched results. We expect that this skew has affected our calculation of Negative Predictive Value of the test and may have falsely inflated this value. Matthew’s Correlation Coefficient attempts to adjust for this skew [25], and yielded a score of 0.37, suggesting that this bias should be considered in the interpretation of these results. It is likely this is due to the wide scope of the testing programme in the context of low prevalence at the time these tests were taken [10,13,23] however many PCR tests in community test centres are also conducted by self-swabbing which may also further bias results where an individual shows poor sampling technique. This bias should be limited since the majority of this study population will likely have received some level of clinical training in their job, and therefore

should have a better understanding of good sampling technique than a member of the general public. The large sample size in this study will also act in opposition to this proposed bias.

Further validation of data would be beneficial on a wider scale, as initial data exploration showed inconsistencies in reported job roles and reasonable demographic information. Regardless of this, the linkage of this data to a care-home via unique organisation number suggests that the tests were likely to have been taken in a domestic care setting and would therefore be conducted in accordance with guidance.

We propose that this study contributes further to the justification of the use of lateral flow testing at times of high prevalence due to the high specificity of this test and high PPV at times of increased prevalence. It is concerning to see such low sensitivity in this analysis; however this should be considered in light of prevalence at the time results for this study were reported, alongside the bias toward asymptomatic cases in this study population leading to low numbers in some subgroups. Public messaging must continue to promote the reliability of lateral flow tests whilst making clear the limited ability of the test to rule out SARS-CoV-2 infection [6,26]. The self-reporting mechanism in place throughout the first two years of the COVID-19 pandemic offered a unique and extensive system by which data from symptomatic and asymptomatic testing streams could be collected separately and collated with regard to the limitations of each of these systems. Wider qualitative study is needed to understand attitudes towards rapid testing, and the reliability of reported results in light of policy change and individual decision making.

Acknowledgements

We would like to thank all staff involved in the curation and supply of data flows used in COVID-19 surveillance in Wales, particularly Gareth John from Digital Health Care Wales, and Helen Clayton and Laura Dexter from Public Health Wales for their work in the

development and upkeep of COVID-19 testing data used in this analysis. Thanks also to Laia Fina for epidemiological in the early stages of this project. Finally, we would like to extend our gratitude to all laboratory staff working in both NHS Wales and Lighthouse labs for their hard work processing COVID-19 tests throughout the pandemic. The author(s) read and approved the final manuscript.

References

1. WHO Director-General's opening remarks at the media briefing on COVID-19. World Health Organisation. 2020. Available from: https://www.who.int/director-general/speeches/detail/who-director-general-s-opening-remarks-at-the-media-briefing-on-covid-19---11-march-2020
2. Coronaviridae Study Group of the International Committee on Taxonomy of Viruses. The species severe acute respiratory syndrome-related coronavirus: Classifying 2019-ncov and naming it SARS-COV-2. Nature Microbiology. 2020;5(4):536–44. doi:10.1038/s41564-020-0695-z
3. Drakesmith M, Collins B, Jones A, Nnoaham K, Thomas DR. Cost-effectiveness of a whole-area testing pilot of asymptomatic SARS-COV-2 infections with lateral flow devices: A modelling and Economic Analysis Study. BMC Health Services Research. 2022;22. doi:10.1186/s12913-022-08511-3
4. Du Z, Pandey A, Bai Y, Fitzpatrick MC, Chinazzi M, Pastore y Piontti A, et al. Comparative cost-effectiveness of SARS-COV-2 testing strategies in the USA: A modelling study. The Lancet Public Health. 2021;6. doi:10.1016/s2468-2667(21)00002-5
5. Guglielmi, G. Fast coronavirus tests: what they can and can’t do. Nature, 2020;585(7826):496-8.
6. Peto T, UK COVID-19 Lateral Flow Oversight Team. Covid-19: Rapid antigen detection for SARS-COV-2 by lateral flow assay: A national systematic evaluation of sensitivity and

specificity for mass-testing. EClinicalMedicine. 2021;36:100924. doi:10.1016/j.eclinm.2021.100924

7. Welsh Government. Testing Strategy for Wales. 2021
8. Welsh Government. Written Statement: Enhancing COVID-19 Testing in Care Homes. 2021
9. Welsh Government. Written Statement: Prioritising PCR Testing. 2022
10. Healy B, Khan A, Metezai H, Blyth I, Asad H. The impact of false positive COVID-19 results in an area of low prevalence. Clinical Medicine. 2020;21. doi:10.7861/clinmed.2020-0839
11. Larremore DB, Wilder B, Lester E, Shehata S, Burke JM, Hay JA, et al. Test sensitivity is secondary to frequency and turnaround time for covid-19 screening. Science Advances. 2021;7. doi:10.1126/sciadv.abd5393
12. Welsh Government Technical Advisory Cell, 2020. Core principles for utilisation of RT-PCR tests for detection of SARS-CoV-2.
13. Office for National Statistics. Coronavirus (COVID-19) Infection Survey: Wales Dataset (2022 Edition). 2022. Available from: https://www.ons.gov.uk/peoplepopulationandcommunity/healthandsocialcare/conditionsanddiseases/datasets/covid19infectionsurveywales/2022
14. Mistry DA, Wang JY, Moeser M-E, Starkey T, Lee LY. A systematic review of the sensitivity and specificity of lateral flow devices in the detection of SARS-COV-2. BMC Infectious Diseases. 2021;21. doi:10.1186/s12879-021-06528-3
15. Mitchell SL, Orris S, Freeman T, Freeman MC, Adam M, Axe M, et al. Performance of SARS-COV-2 antigen testing in symptomatic and asymptomatic adults: A single-center evaluation. BMC Infectious Diseases. 2021;21. doi:10.1186/s12879-021-06716-1
16. UK Government. Asymptomatic testing for SARSCoV-2 using antigen-detecting lateral flow devices: Evidence from performance data October 2020 – May 2021.Department for Health & Social Care; 2021

17. Walsh KA, Jordan K, Clyne B, Rohde D, Drummond L, Byrne P, et al. SARS-COV-2 detection, viral load and infectivity over the course of an infection. Journal of Infection. 2020;81: 357–371. doi:10.1016/j.jinf.2020.06.067
18. He X, Lau EH, Wu P, Deng X, Wang J, Hao X, et al. Temporal Dynamics in viral shedding and transmissibility of COVID-19. Nature Medicine. 2020;26: 672–675. doi:10.1038/s41591-020-0869-5
19. Cevik M, Tate M, Lloyd O, Maraolo AE, Schafers J, Ho A. SARS-COV-2, SARS-COV, and MERS-COV viral load dynamics, duration of viral shedding, and infectiousness: A systematic review and meta-analysis. The Lancet Microbe. 2021;2. doi:10.1016/s2666-5247(20)30172-5
20. Crozier A, Rajan S, Buchan I, McKee M. Put to the test: Use of rapid testing technologies for covid-19. BMJ. 2021; doi:10.1136/bmj.n208
21. Jefferson T, Spencer EA, Brassey J, Heneghan C. Viral cultures for coronavirus disease 2019 infectivity assessment: A systematic review. Clinical Infectious Diseases. 2020;73. doi:10.1093/cid/ciaa1764
22. Welsh Government. Self-isolation Guidance. 2021.
23. Office for National Statistics. Coronavirus (COVID-19) Infection Survey, UK: 18 June 2021. 2021.
24. Amoo OS, Adewara F, Tijani B, Onuigbo TI, Ikemefuna AS, Oraegbu JI, et al. The use of DIY (do it yourself) sampling and telemonitoring model for COVID-19 qPCR testing scale up. PLOS ONE. 2021;16. doi:10.1371/journal.pone.0259398
25. Chicco D, Jurman G. The advantages of the Matthews correlation coefficient (MCC) over F1 score and accuracy in binary classification evaluation. BMC Genomics. 2020;21. doi:10.1186/s12864-019-6413-7
26. Deeks JJ, Raffle AE. Lateral flow tests cannot rule out SARS- COV-2 infection. BMJ. 2020;: m4787. doi:10.1136/bmj.m4787

Figure 1

Click here to access/download;Figure;Fig 1.tif

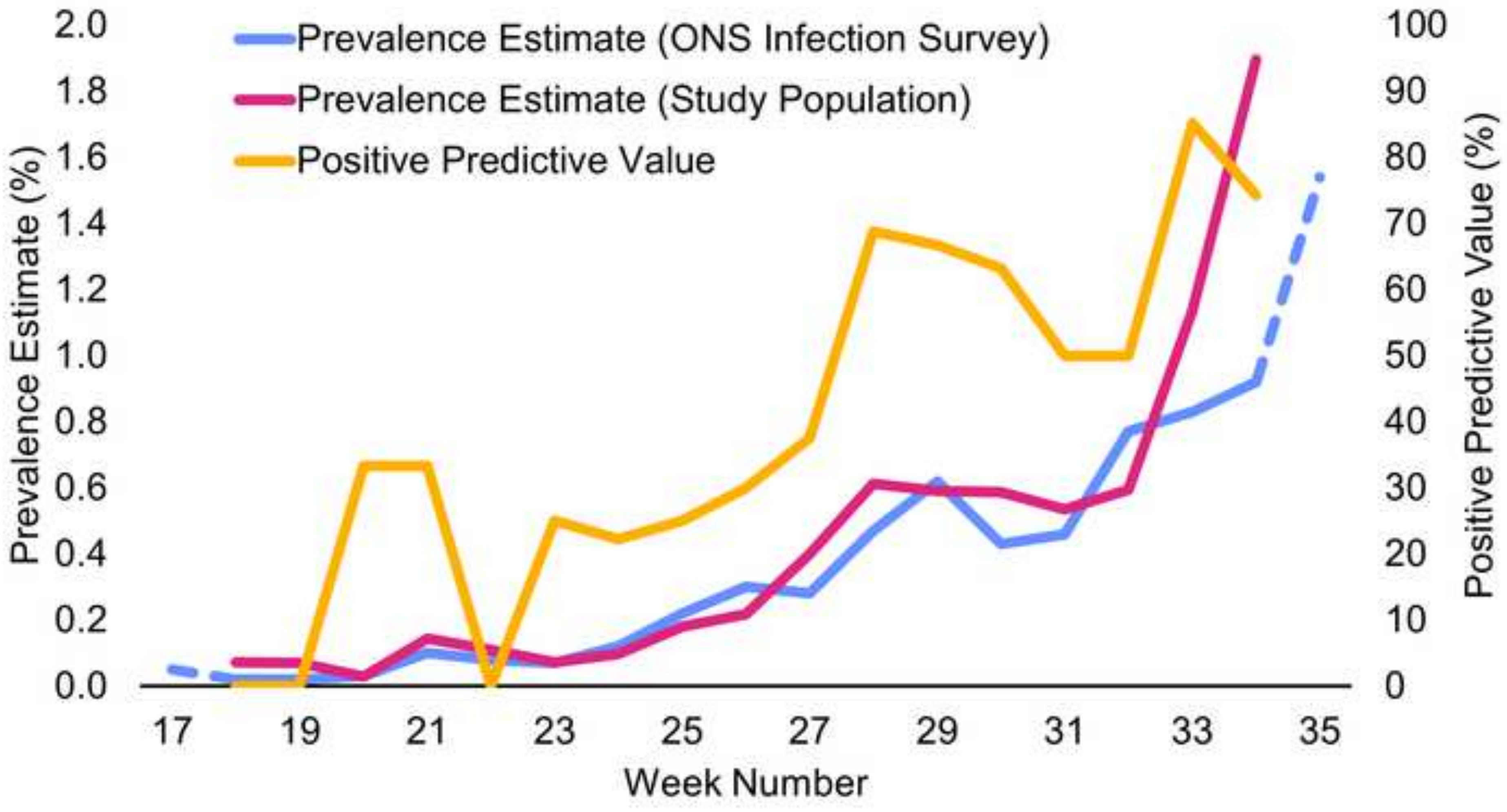

Click here to access/download;Figure;Fig 2.tif

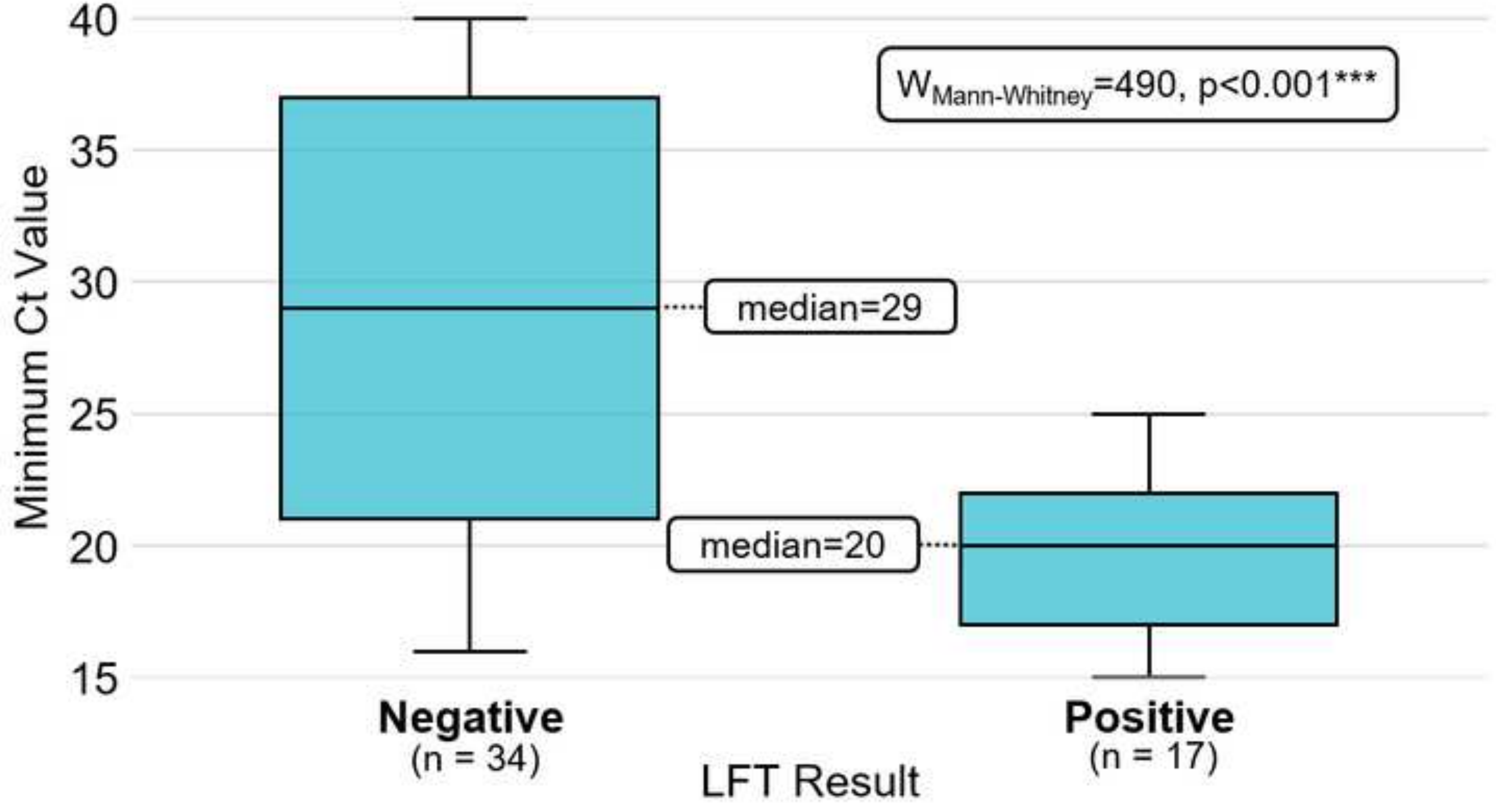

Minimum Dataset

Click here to access/download
Supporting Information
LFT Test Performance Minimum Dataset.xlsx
